# Pomegranate *(Punicagranatum)* juice decreases lipid peroxidation, but has no effect on plasma advanced glycated end-products in adults with type 2 diabetes: a randomized double-blind clinical trial

**DOI:** 10.3402/fnr.v59.28551

**Published:** 2015-09-08

**Authors:** Golbon Sohrab, Pooneh Angoorani, Maryam Tohidi, Hadi Tabibi, Masoud Kimiagar, Javad Nasrollahzadeh

**Affiliations:** 1 Department of Clinical Nutrition and Dietetics, Faculty of Nutrition Sciences and Food Technology, Shahid Beheshti University of Medical Sciences, Tehran, Iran; 2 Prevention of Metabolic Disorders Research Center, Research Institute for Endocrine Sciences, Shahid Beheshti University of Medical Sciences, Tehran, Iran

**Keywords:** human nutrition, metabolism, health claims

## Abstract

**Introduction:**

Diabetes mellitus characterized by hyperglycemia could increase oxidative stress and formation of advanced glycated end-products (AGEs), which contribute to diabetic complications. The purpose of this study was to assess the effect of pomegranate juice (PJ) containing natural antioxidant on lipid peroxidation and plasma AGEs in patients with type 2 diabetes (T2D).

**Materials and methods:**

In a randomized, double-blind, placebo-controlled trial, 44 patients (age range 56±6.8 years), T2D were randomly assigned to one of two groups: group A (PJ, *n*=22) and group B (Placebo, *n*=22). At the baseline and the end of 12-week intervention, biochemical markers including fasting plasma glucose, insulin, oxidative stress, and AGE markers including carboxy methyl lysine (CML) and pentosidine were assayed.

**Results:**

At baseline, there were no significant differences in plasma total antioxidant capacity (TAC) levels between the two groups, but malondialdehyde (MDA) decreased levels were significantly different (*P*<0.001). After 12 weeks of intervention, TAC increased (*P*<0.05) and MDA decreased (*P*<0.01) in the PJ group when compared with the placebo group. However, no significant differences were observed in plasma concentration of CML and pentosidine between the two groups.

**Conclusions:**

The study showed that PJ decreases lipid peroxidation. Therefore, PJ consumption may delay onset of T2D complications related to oxidative stress.

Hyperglycemia in diabetes causes tissue damage through various mechanisms and contributes to diabetic complications (1). Several lines of evidence indicate that these mechanisms are activated by a single upstream event: mitochondrial overproduction of reactive oxygen species (ROS). Therefore, oxidative stress is implicated as a major factor in the development of diabetes complications. Increased formation of advanced glycated end-products (AGEs) is one of the mechanisms that contribute to hyperglycemia's tissue damage (2). Due to hyperglycemia in diabetes, reducing sugars react non-enzymatically with free amino groups of protein to form a diverse group of protein-bound moieties known as AGEs (3). Plasma proteins modified by AGE precursors bind to AGE receptors on cells, such as macrophages, vascular endothelial cells, and vascular smooth muscle cells, and this binding induces the production of ROS, causing multiple pathological changes in gene expression (4).

Some of the best chemically characterized AGEs in humans are pentosidine and carboxy methyl lysine (CML) (5). Peripheral artery disease, a macrovascular complication of hyperglycemia of diabetes, has been strongly associated with serum malondialdehyde (MDA), an indicator of ROS, and with AGEs in type 2 diabetes (T2D) (6). Dietary supplementation, with antioxidant phytochemicals, may be a successful strategy to reduce the risk of pathological complications (7). Pomegranate *(Punicagranatum)* juice (PJ) possesses the highest antioxidant capacity when compared with the commonly consumed polyphenol-rich beverages (8). PJ supplementation has been shown to ameliorate hypertension and reduce risk factors of atherosclerosis in a few clinical studies (9–12), and the effects of PJ consumption on AGEs have not previously been studied. The present randomized clinical trial was designed to explore the effects of 12-week PJ consumption on plasma AGEs (including pentosidine and CML) and oxidative stress on patients with type 2 diabetes.

## Materials and methods

### Patients and study design

This study was a randomized, double-blind, placebo-controlled trial. Forty-four patients (23 men and 21 women, age range 56±6.8 years), at least 1-year post-type 2 diabetes diagnosis, were selected based on their medical records in Iran Charity Foundation for Special Diseases and Health Center in Tehran, Iran. All the patients were taking oral hypoglycemic agents, and none were using insulin. In addition, patients were not smokers and not suffering from any other chronic diseases and, if female, were not taking estrogen or progesterone. At baseline, patients were stratified by gender and randomly assigned to one of the two groups: group A (PJ, *n*=22) and group B (Placebo, *n*=22). Random allocation of patients to treatment groups was performed by sequentially numbered containers. Randomization was performed by an assistant, and the group allocation was blinded for the investigator and participants. Written informed consent was obtained from all the patients. Ethical approval for the trial was obtained from Ethical Committee of National Nutrition and Food Technology Research Institute (Tehran, Iran). This clinical trial has been registered in the Iranian Registry of Clinical Trials at www.irct.ir (ID No: IRCT201206144010N8).

### Intervention and compliance

At baseline, patients were stratified by gender and randomly assigned to consume 250 ml/day PJ, or a control beverage of similar color and energy content for 12 weeks. The study product was packaged in single-serving labeled bottles, so that neither subjects nor staff members were aware of treatment assignment. The subjects were asked not to change their dietary habits, physical activities, or drug regimens. The patients were contacted every week to evaluate compliance to intervention and inquire regarding possible side effects. Each patient was provided with a fixed number of PJ bottles and instructions to return the unused bottles at the end of the study. Based on the number of returned bottles by each patient, their compliance was determined, which was 90%. Patients were excluded from the analysis if they consumed <90% of the packets, had changed their medication, or reported severe side effects. No adverse events were reported.

### Measurements

Each subject's weight was recorded, while wearing light clothing and no shoes, using digital scales to the nearest 100 g. Height was measured to the nearest 0.5 cm. Body mass index (BMI) was then calculated as weight (kg) divided by square of height (m). Dietary intakes of the subjects were assessed, using a 3-day dietary recall (2 weekdays and 1 weekend day) at baseline and at the end of 12 weeks. The patient's diets were analyzed by Nutritionist IV software (N Squared Computing, San Bruno, CA, USA).

### Biochemical analysis

At baseline and the end of 12-week intervention, 10 ml blood was collected from each patient after a 12–14 h overnight fast. Blood samples to which the anti-coagulant was added were centrifuged at 4,000 rpm for 10 min. The plasma samples were separated into aliquots and were frozen at −80°C, until they were assayed.

Fasting plasma glucose (FPG) concentration was assessed using the colorimetry method by commercial kit (Pars Azemoon, Tehran, Iran) and a Selectra 2 auto-analyzer (Vital Scientific, Spankeren, The Netherlands). Hemoglobin A1c (HbA1c) was assessed using the ion exchange chromatography method by commercial kit (Biosystem, Barcelona, Spain). The coefficient of variation (CV) for FPG was 1.3% and HbA1c was 5.1. Plasma concentration of pentosidine and CML was assessed using enzyme immunoassay (ELISA). Pentosidine and CML by related kits (Cusabio Biotech, Wuhan, China) were measured. Plasma concentration of MDA and total antioxidant capacity (TAC) were measured by colorimetry using kits (Cayman, Ann Arbor, USA; and Biocore diagnostics, Hamburg, Germany), respectively. The CV for pentosidine, CML, MDA, and TAC was 6.9, 7.8, 6.4, and 7.3, respectively.

### Pomegranate and placebo juice

To choose the commercial PJ with the highest polyphenol levels, several hand-squeezed and various commercially available juices were analyzed, using the colorimetric assay. The phenols were determined by the Folin–Ciocalteu reagent, using gallic acid as a standard and had 1,946 mg gallic acid equivalent (GAE)/L of PJ (13). Total flavonoid content, measured by the aluminum chloride colorimetric assay, using catechin standard (14), had 345.87 µg catechin equivalent/ml of PJ. The juice was diluted at 1:10 (v:v) to measure TAC, which is based on the inhibition percent of 2,2′-azino-bis(3-ethylbenzothiazoline-6-sulphonic acid), and when compared with bovine serum albumin (BSA) standard curve, it had 7 mmol/L BSA TAC (3). PJ provides 126 kcal, 24 g sugar, and 1 g protein, while placebo beverage had similar color, taste, and energy content (126 kcal, 24 g sugar, and 1 g protein), but colorimetric assays showed that it has no polyphenols. The juice and the placebo were kept at room temperature (<25°C) until opened as recommended by the manufacturer.

### Statistical methods

Statistical analysis of data was performed using the Statistical Package for the Social Sciences (SPSS, Inc., Chicago, IL, USA) for Windows version 16.0. A *χ*^2^ test was used to compare qualitative variables between the two groups. Normality of quantitative parameters was tested using Kolmogorov–Smirnov test. Since all parameters had normal distributions, independent t-tests and paired t-tests were used to compare parameters between and within the groups, respectively. Adjustment for differences in baselines covariates and changes in variables during the study were performed by analysis of covariance (ANCOVA) using general linear models. The results were expressed as mean±SD, and differences were considered significant at *p*≤0.05.

## Results

There was no significant difference in baseline characteristics between the two groups. Anthropometrical factors did not differ between the two groups at the baseline or at the end of week 12 (Table 1). In addition, these factors did not change significantly within the groups during the study. Dietary intakes of participants are shown in Table 2. Energy and carbohydrate intake was different between the two groups.

**Table 1:** Baseline and anthropometric characteristics of patients in the pomegranate juice and placebo groups

Characteristics	Pomegranate (*n*=22)	Placebo (*n*=22)
Age (years)^a^	55±6.7	56.9±6.8
Duration of diabetes (years)^a^	5.8±3.2	6.5±4.3
Weight (kg)	76.9±15	75.2±15
BMI	29.4±3.9	28.6±4.2
Sex		
Men	11 (50%)	12 (54.5%)
Women	11 (50%)	10 (45.5%)
Anti-hypertensive drugs (%)		
ACE-I or ARB	4 (18.2%)	6 (27.3%)
Anti-hyperlipidemic drugs (%)	3 (13.6%)	4 (18.2%)

BMI: body mass index; ACEI: angiotensin converting enzyme inhibitors; ARB: angiotensin receptor blockers.

a Age and duration of diabetes are presented as mean±SD.

**Table 2:** Dietary intakes of the pomegranate juice and placebo groups^a^

Intake	Groups	Baseline	Week 12
Energy (kcal/d)	Pomegranate	1,784±238^b^	1,787±233^b^
	Placebo	2,028±241	2,011±221
Protein (g/d)	Pomegranate	54±12	58±12
	Placebo	59±10	65±9
Carbohydrate (g/d)	Pomegranate	234±35	266±41^b^
	Placebo	232±27	257±37
Fiber (g/d)	Pomegranate	15±4	15±3
	Placebo	16±3	15±4
Fat (g/d)	Pomegranate	73±20	72±19
	Placebo	84±15	83±19
SAFA (g/d)	Pomegranate	16.2±5.5	17.2±6.4
	Placebo	23.7±8.8	21.3±8.2
MUFA (g/d)	Pomegranate	30.7±11	29.8±8.8
	Placebo	32.5±6.1	33.6±8.7
PUFA (g/d)	Pomegranate	21.4±6.7	19.4±4.4
	Placebo	20.5±6.1	21.7±5.9
Cholesterol (mg/d)	Pomegranate	134.3±60.7	136.1±48.6
	Placebo	169.6±78	189.9±58.2
Vitamin E (mg/d)	Pomegranate	22.7±5.9	20.7±4.7
	Placebo	20.5±6.6	22.1±6.6
Vitamin C (mg/d)	Pomegranate	78.7±54.3	77.3±32.9
	Placebo	60.1±32.8	66.6±32.1
Vitamin A (μg/d)	Pomegranate	559.7±478.3	719.9±609
	Placebo	440.8±349.4	359.7±170.2

BMI: body mass index; MUFA: monounsaturated fatty acids; SAFA: saturated fatty acids; PUFA: polyunsaturated fatty acids. All values are presented as mean±SD.

a *n*=22 for all values.

b *P*<0.05 versus the placebo group.

At baseline, there was no significant difference in plasma TAC levels between the two groups, but MDA levels were significantly different (*p*=0.0001). Within the group, analysis showed that levels of MDA and TAC have changed significantly in the PJ group, compared with the baseline (Table 3). Comparison of TAC between the two groups showed higher values in the PJ group when compared with the control group. ANCOVA was performed to compare MDA between two the groups using the baseline MDA values as the covariate. There was a significant difference between the two groups, indicating lower MDA levels in the PJ group.

At baseline, there were no differences in plasma concentrations of CML and pentosidine between the two groups. At the end of the study, no significant changes were found in plasma concentrations of CML or pentosidine between or within the groups.

**Table 3:** Plasma concentrations of oxidative stress and AGE markers, fasting plasma glucose, and HbA1C in the pomegranate juice and placebo groups^a^

Parameters	Groups	Baseline	Week 12	*P*-value	Mean change from baseline
MDA (µmol/L)	Pomegranate	7.6±1.9	5.7±2.1	0.0001	−1.9±1.3
	Placebo	4.3±2.9	5.2±2.2	NS	0.85±2.3
*P*-value (between groups)		0.0001	0.01^b^		
TAC (U/ml)	Pomegranate	22.8±6.4	27.4±6.8	0.001	4.6±5.8
	Placebo	19.8±4.8	20.7±6.6	NS	0.9±4.2
*P*-value (between groups)		NS	0.002		
Carboxy methyl lysine (nmol/ml)	Pomegranate	1.7±0.6	1.5±0.4	NS	−0.2±0.6
	Placebo	1.7±0.3	1.5±0.5	NS	−0.2±0.5
*P*-value (between groups)		NS	NS		
Pentosidine (pmol/ml)	Pomegranate	49±53	59±46	NS	10.2±53.3
	Placebo	79±83	65±46	NS	−14.3±72.5
*P*-value (between groups)		NS	NS		
Fasting plasma glucose (mg/dl)	Pomegranate	160.3±47.8	162±35.2	NS	2±45.3
	Placebo	148.7±42.1	158.6±49.1	NS	9.8±43.3
*P*-value (between groups)		NS	NS		
HbA1C (%)	Pomegranate	8.4±1.2	8.1±1.1	NS	−0.3±0.6
	Placebo	7.5±1.1	7.3±0.1	NS	−0.2±0.4
*P*-value (between groups)		NS	NS		

MDA: malondialdehyde; TAC: total antioxidant capacity; NS: non-significant. All values are presented as mean±SD.

a *n*=22 for all values.

b ANCOVA was performed using baseline values as covariate.

## Discussion

The results of the current study indicate that daily consumption of PJ could increase plasma TAC and decrease plasma MDA in diabetic patients while it has no effect on AGE levels. Juice extracted from the pomegranate has been shown to have the greatest *in vitro* antioxidant potency among commonly consumed beverages (8, 15). It is worth mentioning that the baseline MDA levels in the PJ group were higher than in the placebo group. Therefore, in addition to antioxidative properties of PJ that could influence plasma MDA level, part of the decline in MDA levels following PJ consumption could be due to the higher baseline levels of MDA in the PJ group. Increased serum TAC and paraoxonase and decreased LDL sensitivity to oxidation have been reported in healthy persons (11) and patients with carotid arterial stenosis following PJ consumption (16). Pomegranate is known as a very rich source of anthocyanins, ellagic acid, punicalagins, catechins, and gallocatechins (15, 17, 18). Anthocyanin-rich beverage ingestion has been reported to decrease plasma and urinary concentrations of MDA and to improve plasma antioxidant capacity in healthy female volunteers (19). Furthermore, ellagic acid has been shown to reduce MDA levels in the brain of streptozotocin-induced diabetic rats (20). Consumption of pomegranate polyphenolic extract has led to a significant decrease in serum MDA in diabetic patients (21). In addition, the juice has been shown to decrease serum lipid peroxides and increase paraoxonase (22). Although polyphenolic extract of PJ is effective in reducing oxidative stress, the extract is less potent than the juice, which may indicate that other factors in the juice (unique sugars) may contribute to mitigate oxidative stress (22). The mechanism by which PJ or its compounds could ameliorate lipid peroxidation and oxidative stress is not well known but might occur by directly neutralizing the generated reactive oxygen species (23), increasing certain antioxidant enzyme activities such as paraoxonase (22), and inhibiting or activating certain transcriptional factors, such as nuclear factor *κ*B (24, 25) and peroxisome proliferator-activated receptor *γ* (26). Interestingly, a recent study showed that administration of pomegranate fruit extract for seven consecutive days before and after methotrexate challenge in Swiss albino mice reduced ROS generation in hepatocytes mainly by differential regulation of the activation of the transcription factors nuclear factor (erythroid-derived 2)-like 2 and nuclear factor *κ*B as a consequence of which the antioxidant defense mechanism in the liver was upregulated (24).

Protein glycation and AGE formation are the result of non-enzymatic reactions with glucose; AGE level has previously correlated with HbA1c levels (27, 28). Serum levels of pentosidine and CML have been reported to be higher in patients with type 2 diabetes, compared with non-diabetic controls (29). In addition, during the process of AGE formation, ROS are produced as by-products of the late steps of glycation reactions, and these radicals in turn further promote glycation (30). AGE levels have been associated with ROS in diabetic patients (31). Previously, *in vitro* and animal studies have shown that polyphenolic extracts from a different source could inhibit AGE formation (32, 33). In our study, although we found that PJ resulted in a significant improvement in TAC an MDA status, there was no significant difference between the PJ group and the placebo group regarding plasma concentration of pentosidine and CML. One possible explanation for this finding is that the patients in the current study did not have such elevated baseline AGE levels. In a study by Lapolla et al., plasma pentosidine levels of a healthy subject were 63.2 pmol/ml which are comparable to the pentosidine levels of our study subjects, while in diabetic patients without peripheral artery disease pentosidine levels were 85.5 pmol/ml and in those with peripheral artery disease were 109.2 pmol/ml (6). Since no significant difference were found in glycemic control between the two groups, comparable non-enzymatic reaction with glucose and protein glycation could have occurred in both the groups. Another possibility is that to influence plasma AGEs, taking antioxidant source should continue for a more prolonged time. In a study by Shimada et al., antioxidant therapy for 6 months significantly decreased hemoglobin carboxymethyl valine residue levels, though HbA1c did not change (34).

In the current study, although dietary energy and carbohydrate intake differed between the two groups, no significant changes in body weight, plasma glucose, or HbA1c were observed between the two groups.

Some limitations of the present study are that PJ consumption was relatively short in duration and few parameters reflecting oxidative stress and advanced glycation process were measured.

In conclusion, 12-week consumption of PJ does not influence plasma levels of the AGEs, pentosidine and CML, in patients with T2D while it improved plasma TAC an MDA status. PJ contain simple sugars. However, its consumption did not impair glycemic control of diabetic patients. Therefore, it could be potential natural drinks in diabetic diet with favorable properties.

## References

[1] Brownlee M (2005). The pathobiology of diabetic complications: a unifying mechanism. Diabetes.

[2] Giacco F, Brownlee M (2010). Oxidative stress and diabetic complications. Circ Res.

[3] Singh R, Barden A, Mori T, Beilin L (2001). Advanced glycation end-products: a review. Diabetologia.

[4] Goldin A, Beckman JA, Schmidt AM, Creager MA (2006). Advanced glycation end products: sparking the development of diabetic vascular injury. Circulation.

[5] Goh SY, Cooper ME (2008). Clinical review: the role of advanced glycation end products in progression and complications of diabetes. J Clin Endocrinol Metab.

[6] Lapolla A, Piarulli F, Sartore G, Ceriello A, Ragazzi E, Reitano R (2007). Advanced glycation end products and antioxidant status in type 2 diabetic patients with and without peripheral artery disease. Diabetes Care.

[7] Dembinska-Kiec A, Mykkanen O, Kiec-Wilk B, Mykkanen H (2008). Antioxidant phytochemicals against type 2 diabetes. Br J Nutr.

[8] Seeram NP, Aviram M, Zhang Y, Henning SM, Feng L, Dreher M (2008). Comparison of antioxidant potency of commonly consumed polyphenol-rich beverages in the United States. J Agric Food Chem.

[9] Aviram M, Dornfeld L (2001). Pomegranate juice consumption inhibits serum angiotensin converting enzyme activity and reduces systolic blood pressure. Atherosclerosis.

[10] Rosenblat M, Volkova N, Aviram M (2010). Pomegranate juice (PJ) consumption antioxidative properties on mouse macrophages, but not PJ beneficial effects on macrophage cholesterol and triglyceride metabolism, are mediated via PJ-induced stimulation of macrophage PON2. Atherosclerosis.

[11] Aviram M, Dornfeld L, Rosenblat M, Volkova N, Kaplan M, Coleman R (2000). Pomegranate juice consumption reduces oxidative stress, atherogenic modifications to LDL, and platelet aggregation: studies in humans and in atherosclerotic apolipoprotein E-deficient mice. Am J Clin Nutr.

[12] Aviram M, Volkova N, Coleman R, Dreher M, Reddy MK, Ferreira D (2008). Pomegranate phenolics from the peels, arils, and flowers are antiatherogenic: studies in vivo in atherosclerotic apolipoprotein e-deficient (E 0) mice and *in vitro* in cultured macrophages and lipoproteins. J Agric Food Chem.

[13] Bray HG, Thorpe WV (1954). Analysis of phenolic compounds of interest in metabolism. Methods Biochem Anal.

[14] Hajimahmoodi M, Oveisi MR, Sadeghi N, Jannat B, Hadjibabaie M, Farahani E (2008). Antioxidant properties of peel and pulp hydro extract in ten Persian pomegranate cultivars. Pak J Biol Sci.

[15] Gil MI, Tomas-Barberan FA, Hess-Pierce B, Holcroft DM, Kader AA (2000). Antioxidant activity of pomegranate juice and its relationship with phenolic composition and processing. J Agric Food Chem.

[16] Aviram M, Rosenblat M, Gaitini D, Nitecki S, Hoffman A, Dornfeld L (2004). Pomegranate juice consumption for 3 years by patients with carotid artery stenosis reduces common carotid intima-media thickness, blood pressure and LDL oxidation. Clin Nutr.

[17] Alighourchi H, Barzegar M, Abbasi S (2007). Anthocyanins characterisation of 15 Iranian pomegranate (*Punica granatum* L.) varieties and their variation after cold storage and pasteurisation. Eur Food Res Technol.

[18] de Pascual-Teresa S, Santos-Buelga C, Rivas-Gonzalo JC (2000). Quantitative analysis of flavan-3-ols in Spanish foodstuffs and beverages. J Agric Food Chem.

[19] Kuntz S, Kunz C, Herrmann J, Borsch CH, Abel G, Fröhling B (2014). Anthocyanins from fruit juices improve the antioxidant status of healthy young female volunteers without affecting anti-inflammatory parameters: results from the randomised, double-blind, placebo-controlled, cross-over ANTHONIA (ANTHOcyanins in Nutrition Investigation Alliance) study. Br J Nutr.

[20] Uzar E, Alp H, Cevik MU, FIrat U, Evliyaoglu O, Tufek A (2012). Ellagic acid attenuates oxidative stress on brain and sciatic nerve and improves histopathology of brain in streptozotocin-induced diabetic rats. Neurol Sci.

[21] Basu A, Newman ED, Bryant AL, Lyons TJ, Betts NM (2012). Pomegranate polyphenols lower lipid peroxidation in adults with type 2 diabetes but have no effects in healthy volunteers: a pilot study. J Nutr Metab.

[22] Rosenblat M, Hayek T, Aviram M (2006). Anti-oxidative effects of pomegranate juice (PJ) consumption by diabetic patients on serum and on macrophages. Atherosclerosis.

[23] Banihani S, Swedan S, Alguraan Z (2013). Pomegranate and type 2 diabetes. Nutr Res.

[24] Mukherjee S, Ghosh S, Choudhury S, Adhikary A, Manna K, Dey S (2013). Pomegranate reverses methotrexate-induced oxidative stress and apoptosis in hepatocytes by modulating Nrf2-NF-kappaB pathways. J Nutr Biochem.

[25] Aggarwal BB, Shishodia S (2004). Suppression of the nuclear factor-kappaB activation pathway by spice-derived phytochemicals: reasoning for seasoning. Ann N Y Acad Sci.

[26] Shiner M, Fuhrman B, Aviram M (2007). Macrophage paraoxonase 2 (PON2) expression is up-regulated by pomegranate juice phenolic anti-oxidants via PPAR gamma and AP-1 pathway activation. Atherosclerosis.

[27] Galler A, Muller G, Schinzel R, Kratzsch J, Kiess W, Munch G (2003). Impact of metabolic control and serum lipids on the concentration of advanced glycation end products in the serum of children and adolescents with type 1 diabetes, as determined by fluorescence spectroscopy and nepsilon-(carboxymethyl)lysine ELISA. Diabetes Care.

[28] Garay-Sevilla ME, Regalado JC, Malacara JM, Nava LE, Wrobel-Zasada K, Castro-Rivas A (2005). Advanced glycosylation end products in skin, serum, saliva and urine and its association with complications of patients with type 2 diabetes mellitus. J Endocrinol Invest.

[29] Ghanem AA, Elewa A, Arafa LF (2011). Pentosidine and N-carboxymethyl-lysine: biomarkers for type 2 diabetic retinopathy. Eur J Ophthalmol.

[30] Hunt JV, Bottoms MA, Mitchinson MJ (1993). Oxidative alterations in the experimental glycation model of diabetes mellitus are due to protein–glucose adduct oxidation. Some fundamental differences in proposed mechanisms of glucose oxidation and oxidant production. Biochem J.

[31] Gupta A, Tripathi AK, Tripathi RL, Madhu SV, Banerjee BD (2007). Advanced glycosylated end products-mediated activation of polymorphonuclear neutrophils in diabetes mellitus and associated oxidative stress. Indian J Biochem Biophys.

[32] Peng CH, Chyau CC, Chan KC, Chan TH, Wang CJ, Huang CN (2011). *Hibiscus sabdariffa* polyphenolic extract inhibits hyperglycemia, hyperlipidemia, and glycation-oxidative stress while improving insulin resistance. J Agric Food Chem.

[33] Jariyapamornkoon N, Yibchok-anun S, Adisakwattana S (2013). Inhibition of advanced glycation end products by red grape skin extract and its antioxidant activity. BMC Complement Altern Med.

[34] Shimada S, Tanaka Y, Ohmura C, Tamura Y, Shimizu T, Uchino H (2005). N-(carboxymethyl)valine residues in hemoglobin (CMV-Hb) reflect accumulation of oxidative stress in diabetic patients. Diabetes Res Clin Prac.

